# CT-Derived Radiomic Features for the Non-Invasive Differentiation of Mediastinal Lymphadenopathy in Lung Cancer and Sarcoidosis

**DOI:** 10.3390/biomedicines14061327

**Published:** 2026-06-11

**Authors:** Demet Doğan, Coşku Öksüz, Özgür Çakır, Zuhal Güllü, Oğuzhan Urhan

**Affiliations:** 1 Department of Radiology, Faculty of Medicine, İstanbul Okan University, İstanbul 34947, Türkiye; 2 Department of Electrical and Electronics Engineering, Izmir Bakircay University, Izmir 35665, Türkiye; cosku.oksuz@bakircay.edu.tr; 3 Department of Radiology, Faculty of Medicine, Kocaeli University, Kocaeli 41001, Türkiye; cakirozgur@hotmail.com; 4 Department of Pulmonology, Faculty of Medicine, İstanbul Okan University, İstanbul 34947, Türkiye; zuhalgullu@gmail.com; 5 Department of Electronics & Telecommunication Engineering, Kocaeli University, Kocaeli 41001, Türkiye; urhano@kocaeli.edu.tr

**Keywords:** sarcoidosis, mediastinum, lymphadenopathy, lung neoplasms, multidetector computed tomography, radiomics

## Abstract

**Background/Objectives:** Differentiating mediastinal lymphadenopathy associated with lung cancer from sarcoidosis remains a clinical challenge because of overlapping imaging findings. This study evaluated whether CT-derived radiomic features, alone and in combination with clinical variables, could support the non-invasive differentiation of these two entities. **Methods:** In this retrospective single-center study, 204 histopathologically confirmed mediastinal lymph nodes were analyzed. A total of 107 radiomic features were extracted from manually segmented contrast-enhanced thoracic CT images. Multiple feature selection methods, dimensionality reduction techniques, and machine learning classifiers were evaluated using patient-level five-fold cross-validation. Additional clinical-only, combined clinical–radiomic, one-node-per-patient sensitivity, and exploratory interobserver feature stability analyses were performed. **Results:** Among radiomics-only models, LASSO achieved the highest ROC–AUC of 0.9108, whereas ElasticNet achieved the highest accuracy of 81.20%. The clinical-only ensemble model using age, sex, and smoking status showed strong performance, with an accuracy of 94.92% and an ROC–AUC of 0.9733. Selected combined clinical–radiomic models showed numerically higher performance; the combined correlation-filtered ensemble model achieved the highest accuracy of 97.78% and an ROC–AUC of 1.0000. Clinical integration also yielded more compact feature subsets in some methods, as combined LASSO selected 9.6 variables while improving ROC–AUC from 0.9108 to 0.9667 compared with radiomics-only LASSO. In the one-node-per-patient sensitivity analysis, clinical-only and combined models retained high performance, whereas radiomics-only LASSO showed reduced performance. Exploratory interobserver analysis showed moderate reproducibility for only a subset of radiomic features. **Conclusions:** CT-derived radiomic features may provide complementary information for differentiating mediastinal lymphadenopathy associated with lung cancer from that associated with sarcoidosis. However, clinical variables were highly informative, and the incremental value of radiomics should be interpreted cautiously. Further multicenter studies with external validation, standardized segmentation protocols, and clinically balanced cohorts are required before routine clinical implementation can be recommended.

## 1. Introduction

Sarcoidosis is a multisystem disease of unknown etiology, characterized by non-caseating granulomatous inflammation, most commonly involving the lungs and mediastinal lymph nodes [1,2]. Lung cancer remains one of the leading causes of cancer-related mortality worldwide [3], and mediastinal lymph node involvement plays a critical role in staging, prognosis, and treatment planning [4]. Both conditions may present with mediastinal lymphadenopathy, posing a significant diagnostic challenge.

In the evaluation of mediastinal lymphadenopathy, clinical findings, laboratory tests, radiologic imaging, and pathological examination are commonly used [5]. Contrast-enhanced thoracic computed tomography (CT) provides valuable information regarding lymph node size, shape, margins, internal structure, and enhancement patterns. However, conventional CT parameters, including lymph node size and attenuation, often have limited discriminative value [6], as lymph nodes affected by sarcoidosis may closely resemble metastatic lymph nodes in lung cancer [7]. Consequently, reliable differentiation based solely on conventional imaging findings remains difficult in routine clinical practice.

Similarly, increased 18F-FDG uptake may be observed in both conditions on PET/CT, limiting its specificity despite high sensitivity [8]. In clinical practice, invasive diagnostic procedures, including transbronchial needle aspiration, mediastinoscopy, and endobronchial ultrasound (EBUS)-guided biopsy, are frequently required to establish a definitive diagnosis [9,10]. However, these approaches are associated with limitations such as sampling insufficiency, procedure-related risks, patient discomfort, and increased healthcare costs. These challenges highlight the need for accurate, non-invasive, and objective diagnostic tools.

Radiomics, which enables the extraction of high-dimensional quantitative features from medical images, has emerged as a promising approach to address such diagnostic limitations. By capturing imaging patterns related to tissue heterogeneity and morphology, radiomic analyses have demonstrated clinical value in lung cancer diagnosis and staging [11,12], as well as in various benign–malignant differentiation tasks [13]. However, existing studies have predominantly focused on tumor characterization or differentiation between other disease entities, and data specifically addressing mediastinal lymphadenopathy in sarcoidosis and lung cancer remain limited.

In this study, we aimed to evaluate whether CT-based radiomic features can differentiate mediastinal lymphadenopathy associated with lung cancer from that related to sarcoidosis. We further investigated whether radiomic models could provide complementary information for non-invasive clinical decision-making

## 2. Materials and Methods

### 2.1. Study Design and Population

This retrospective, single-center study was conducted at a tertiary care university hospital and approved by the local institutional ethics committee (Meeting Date: 11 February 2026; Meeting Number: 198; Decision No: 14). All procedures were conducted in accordance with the Declaration of Helsinki and relevant national data protection regulations. The requirement for informed consent was waived due to the retrospective design and anonymized data use, in accordance with national and institutional regulations.

The study population included adult patients who underwent contrast-enhanced chest CT and had histopathologically confirmed mediastinal lymphadenopathy related to lung cancer or sarcoidosis. Patients with a history of other malignancies, active infection, prior thoracic surgery, or inadequate image quality that precluded reliable lymph node segmentation were excluded from the study. Only clearly identifiable mediastinal lymph nodes suitable for reliable segmentation were included in the analysis. For the primary radiomic modeling analysis, manual segmentation was performed by a radiologist with 15 years of experience in thoracic imaging to ensure consistency in ROI delineation.

Radiomic features were extracted at the lymph-node level. In patients with multiple eligible lymph nodes, all lymph nodes meeting the segmentation quality criteria were included in the analysis. In total, 204 mediastinal lymph nodes were analyzed, including 102 lymph nodes associated with sarcoidosis-related lymphadenopathy and 102 lymph nodes associated with lung cancer-related lymphadenopathy. The overall average number of lymph nodes per patient was 5.51. The average number of lymph nodes per patient was 5.67 in the sarcoidosis group and 5.37 in the lung cancer group. To address the potential non-independence of multiple lymph nodes obtained from the same patient, all cross-validation procedures were performed at the patient level, ensuring that lymph nodes from the same patient were assigned to the same fold. In addition to radiomic analysis, baseline clinical and imaging parameters, including age, sex, smoking status, and lymph node size (short- and long-axis diameters), were recorded and compared between groups, as summarized in Table 1.

### 2.2. CT Imaging Protocol

All CT examinations were performed using a 64-slice multidetector CT scanner (Optima CT 660, General Electric Medical Systems, Milwaukee, WI, USA). Scans were obtained with patients in the supine position. Scanning parameters included a tube voltage of 120 kV and a tube current ranging from 70 to 120 mAs. All images used for radiomic feature extraction were reconstructed with a slice thickness of 1.25 mm.

Contrast-enhanced CT imaging was performed using a water-soluble, non-ionic iodinated contrast agent (300–350 mg I/mL). The contrast material was administered intravenously via an 18-gauge cannula placed in the antecubital vein using an automatic dual-head power injector at a dose of approximately 1 mL/kg (total volume 90–100 mL) and an injection rate of 3–5 mL/s. Image acquisition was initiated after contrast administration according to the standard chest CT protocol. All CT images used in the primary analysis were retrospectively reviewed by an experienced radiologist (D.D.).

### 2.3. Dataset

In this study, radiomic features extracted from contrast-enhanced CT images were used for analysis. CT images containing clinically relevant, manually segmented mediastinal lymph nodes were evaluated. For each lymph node, radiomic features were extracted in three dimensions from manually delineated volumes of interest.

Each lymph node was labeled according to the corresponding clinical diagnosis as either lung cancer-related lymphadenopathy (CA-LAP) or sarcoidosis-related lymphadenopathy (Sarcoidosis-LAP). Accordingly, the classification task was formulated as a binary classification problem.

The final dataset included 204 mediastinal lymph node samples. Each row represented a lymph-node-level feature vector containing 107 radiomic features. Manual segmentation and radiomic feature extraction were performed using 3D Slicer (version 5.8.1; Brigham and Women’s Hospital, Boston, MA, USA) and PyRadiomics (version 3.1.0; Harvard Medical School, Boston, MA, USA), respectively. The extracted features included first-order statistical descriptors, shape-based morphological descriptors, and texture-based matrix features from the GLCM, GLRLM, GLSZM, GLDM, and NGTDM families, consistent with the Image Biomarker Standardisation Initiative (IBSI) recommendations.

### 2.4. Data Preprocessing

Records with missing values, incomplete radiomic feature vectors, or undefined class labels were removed from the dataset. Missing data primarily included incomplete radiomic feature extraction due to segmentation errors or inadequate image quality, as well as undefined class labels required for classification. To ensure model reliability, cases with incomplete radiomic feature vectors or undefined class labels were excluded from the analysis.

Because radiomic features were measured on different numerical scales, z-score normalization was applied. To prevent data leakage, all preprocessing steps, including normalization, were performed within the cross-validation framework using only the training data of each fold. The normalization parameters estimated from the training subset were then applied to the corresponding validation subset.

### 2.5. Feature Selection

#### 2.5.1. Filter-Based Feature Selection

##### Correlation-Based Feature Selection

To reduce multicollinearity among radiomic features, correlation-based feature filtering was applied [14,15]. Pearson correlation coefficients were calculated for all feature pairs, and highly correlated features were identified using predefined correlation thresholds. For each correlated pair, one feature was removed to reduce redundancy. This procedure was performed within each training fold to prevent information leakage. The filtering procedure was repeated using correlation thresholds of 0.6, 0.7, 0.8, and 0.9, resulting in average feature subsets of 17, 22, 34, and 52 radiomic features across the patient-level cross-validation folds, respectively, in addition to the original dataset containing 107 radiomic features.

#### 2.5.2. Embedded Feature Selection

Embedded feature selection methods perform feature selection during model training, allowing relevant variables to be identified while constructing the predictive model. These approaches help reduce redundant features, control model complexity, and decrease the risk of overfitting. In this study, three embedded feature selection methods—LASSO, ElasticNet, and Boruta—were applied to identify radiomic features contributing most to classification performance.

#### LASSO

LASSO (Least Absolute Shrinkage and Selection Operator) is an embedded feature selection method that applies an L1 penalty to regression coefficients, enabling simultaneous feature selection and regularization [16]. By shrinking some coefficients to zero, LASSO automatically removes irrelevant features from the model. Due to its effectiveness in high-dimensional datasets with multicollinearity, LASSO was applied to identify informative radiomic features in this study.

#### ElasticNet

ElasticNet combines the properties of LASSO and Ridge regression by applying both L1 and L2 penalties to regression coefficients [17]. This approach allows correlated features to be retained while performing feature selection. ElasticNet was therefore used in this study to obtain a more stable feature subset in the high-dimensional radiomic dataset.

#### Boruta

Boruta is a wrapper-based feature selection method built on the Random Forest algorithm that aims to identify all relevant features [18]. The method evaluates the importance of each feature by comparing it with randomly permuted shadow features. Features that demonstrate significantly higher importance than the shadow features are retained for the model.

#### 2.5.3. Dimensionality Reduction

Dimensionality reduction techniques were applied to reduce redundancy in the high-dimensional radiomic feature space. In this study, Partial Least Squares (PLS) [19] and Principal Component Regression (PCR) [20] were evaluated. PCR incorporates components derived from principal component analysis (PCA), whereas PLS constructs components by maximizing the covariance between predictor variables and the target variable. The optimal number of components for each method was determined using cross-validation, and their classification performances were subsequently evaluated.

#### 2.5.4. Wrapper-Based Feature Selection

Recursive Feature Elimination (RFE) [21] is a wrapper-based feature selection method that iteratively removes the least important features based on model performance. The model is initially trained using all features, and features contributing least to the classification task are progressively eliminated. In this study, RFE was applied to generate different feature subsets, and the optimal configuration was determined based on classification performance.

### 2.6. Classification

Different machine learning classifiers were evaluated using the complete or selected radiomic feature sets. These included support vector machines (SVM), decision trees, ensemble learning methods, k-nearest neighbor (kNN), neural networks (NN), binary logistic regression, and discriminant analysis models. Classifier training and evaluation were performed within the patient-level cross-validation framework. Model-specific hyperparameters were optimized using Bayesian optimization, as described in Section 2.7.

### 2.7. Hyperparameter Optimization

To improve model performance while minimizing optimistic bias, model-specific hyperparameters were optimized separately within the training subset of each patient-level cross-validation fold. Predefined parameter grids and internal cross-validation were used for model tuning. For k-nearest neighbor models, the number of neighbors was selected from candidate values. For correlation-based filtering, predefined correlation thresholds of 0.6, 0.7, 0.8, and 0.9 were evaluated. For LASSO and ElasticNet, regularization parameters were selected within the training data, and for PCR and PLS, the optimal number of components was determined within the same internal validation framework. For RFE, different feature subset sizes were evaluated, whereas Boruta was applied using Random Forest-based feature importance assessment.

In each outer fold, the validation subset was kept completely unseen during preprocessing, feature selection, dimensionality reduction, hyperparameter tuning, and model development. The optimized model was subsequently evaluated on the held-out validation fold. This fold-specific optimization strategy was used to reduce the risk of data leakage and overly optimistic performance estimation.

### 2.8. Statistical Analysis

Model performance was evaluated using accuracy, recall (sensitivity), specificity, precision, F1-score, and the area under the receiver operating characteristic curve (ROC–AUC). As described above, all models were assessed using patient-level five-fold cross-validation, in which all lymph nodes from the same patient were kept within the same fold. For each fold, preprocessing, feature normalization, feature selection, dimensionality reduction, and hyperparameter optimization were performed using only the training data, and the held-out validation subset was not used during any model development step.

Model performance was calculated on the validation subset of each fold and reported as the mean across the five folds. Multiple feature selection and dimensionality reduction strategies were evaluated within the same validation framework to compare the effect of different feature representations. Recall and ROC–AUC were particularly emphasized because of their clinical relevance in identifying positive cases and assessing discriminative performance. When reported, 95% confidence intervals were estimated across the five cross-validation folds to quantify the uncertainty associated with model performance.

To quantify the incremental value of radiomic features beyond routinely available clinical information, additional multivariable clinical-only and combined clinical–radiomic models were developed using age, sex, and smoking status. In clinical-only analysis, these three variables were used as predictors. In the combined clinical–radiomic analysis, clinical variables were incorporated into the candidate feature space together with radiomic features, and feature selection or dimensionality reduction was repeated within the same patient-level five-fold cross-validation framework. The performance of clinical-only, radiomics-only, and combined clinical–radiomic models was compared primarily using accuracy and ROC–AUC.

To assess whether model performance was influenced by patients contributing multiple lymph nodes, an additional one-node-per-patient sensitivity analysis was performed. In this analysis, one lymph node was randomly selected from each patient because no predefined clinical priority or anatomical hierarchy was available for selecting a single representative node. This random selection procedure was repeated 100 times. Radiomics-only, clinical-only, and combined clinical–radiomic models were re-evaluated under the same patient-level five-fold cross-validation framework, and performance values were summarized as mean ± 95% confidence interval across repetitions.

In addition, an exploratory interobserver radiomic feature stability analysis was performed to assess the reproducibility of radiomic measurements between two radiologists. The analysis included 30 matched cases for which corresponding measurements from both radiologists were available. Radiomic features were extracted separately from both measurement sets, and feature-level reproducibility was assessed using the intraclass correlation coefficient ICC(2,1). Because nodal-location information was not consistently available in the second measurement set, this analysis was interpreted as exploratory rather than definitive node-level reproducibility testing. A log1p-transformed ICC analysis was also performed to reduce the influence of highly skewed radiomic feature distributions.

## 3. Results

In this study, a total of 204 mediastinal lymph nodes were analyzed. Baseline demographic and clinical characteristics are summarized in Table 1.

**Figure 1:**
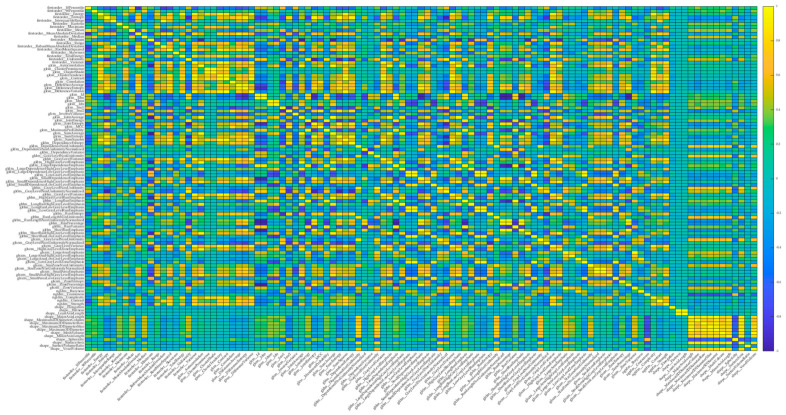
Correlation heatmap of the original radiomic feature set.

A total of 107 radiomic features extracted from contrast-enhanced chest CT images were used to differentiate mediastinal lymphadenopathy associated with lung cancer (CA-LAP) from sarcoidosis-related mediastinal lymphadenopathy (Sarcoidosis-LAP). A comprehensive evaluation of feature selection, dimensionality reduction, and machine learning-based classification strategies was performed within a unified cross-validation framework. Correlation analysis of the original radiomic feature set demonstrated a high degree of inter-feature dependency, indicating substantial redundancy within the high-dimensional radiomic feature space (Figure 1).

The heatmap illustrates the Pearson correlation coefficients among the 107 radiomic features prior to dimensionality reduction, demonstrating substantial inter-feature correlations and redundancy within the high-dimensional radiomic feature space.

Table 2 summarizes the classification performance of machine learning models trained using all 107 radiomic features without dimensionality reduction under patient-level five-fold cross-validation. Logistic regression achieved the highest accuracy, specificity, precision, and F1-score among the evaluated classifiers, with values of 73.5%, 75.0%, 75.3%, and 72.2%, respectively. The ensemble model showed a comparable recall value of 74.0%, whereas the neural network model achieved the highest ROC–AUC of 0.8142. These findings indicate that the full radiomic feature set did not produce a single uniformly superior classifier across all performance metrics. Therefore, reduced-feature and regularized models were further evaluated to determine whether more compact and interpretable feature representations could provide comparable or improved discriminative performance.

After evaluating the classification performance using the complete set of 107 radiomic features, correlation-based feature filtering was applied to examine whether a reduced and less redundant feature space could preserve discriminative performance. Table 3 summarizes the best-performing classifier for each correlation-based feature filtering configuration under patient-level five-fold cross-validation. Depending on the correlation threshold, the radiomic feature space was reduced to 17, 22, 34, and 52 features. Among these configurations, the highest overall performance was obtained with the |ρ| ≥ 0.8 threshold, where the ensemble classifier achieved an accuracy of 75.8%, recall of 79.0%, specificity of 73.3%, precision of 81.3%, F1-score of 73.8%, and ROC–AUC of 0.85. The |ρ| ≥ 0.6 and |ρ| ≥ 0.9 configurations also produced comparable ROC–AUC values of 0.8433 and 0.8425, respectively. These findings indicate that correlation-based feature filtering reduced feature dimensionality while maintaining discriminative performance, with the 34-feature ensemble model providing the most balanced performance across the main evaluation metrics.

In addition to correlation-based filtering, embedded feature selection methods were evaluated to identify radiomic feature subsets that could improve model compactness while preserving or enhancing classification performance. Table 4 summarizes the classification performance obtained using LASSO, ElasticNet, and Boruta under patient-level five-fold cross-validation. ElasticNet selected an average of 42 radiomic features and achieved the highest accuracy, recall, and F1-score among the embedded methods, with values of 81.2%, 87.0%, and 81.6%, respectively. LASSO selected a smaller feature subset, with an average of 26 radiomic features, and achieved the highest ROC–AUC value of 0.9108, indicating strong discriminative performance. In contrast, Boruta retained the most compact feature subset, with eight radiomic features, but demonstrated lower classification performance, with an accuracy of 61.3% and an ROC–AUC of 0.7250. These findings suggest that regularized embedded methods, particularly LASSO and ElasticNet, provided a more favorable balance between feature reduction and classification performance in the patient-level validation framework.

Dimensionality reduction methods were also evaluated to determine whether latent component-based representations could preserve classification performance while reducing the original radiomic feature space. Table 5 summarizes the classification performance obtained using PLS and PCR under patient-level five-fold cross-validation. PLS retained an average of five components and achieved the highest recall among the two dimensionality reduction methods, with a value of 87.00%. In contrast, PCR retained an average of 19 components and achieved higher accuracy, specificity, precision, and ROC–AUC, with values of 79.70%, 81.67%, 81.00%, and 0.8942, respectively. These findings indicate that PCR provided stronger overall discriminative performance, whereas PLS produced a more compact component representation with higher sensitivity.

### 3.1. Feature Selection Frequency Analysis

**Figure 2:**
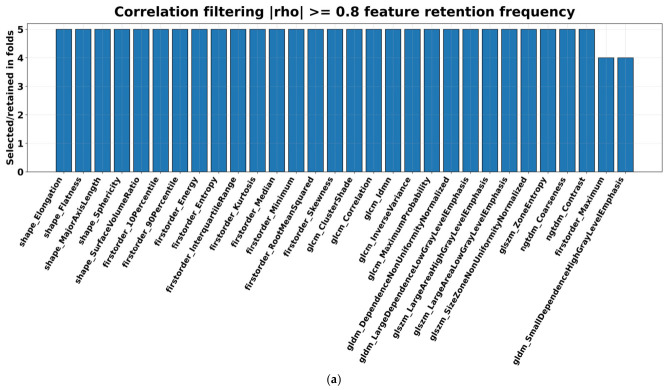

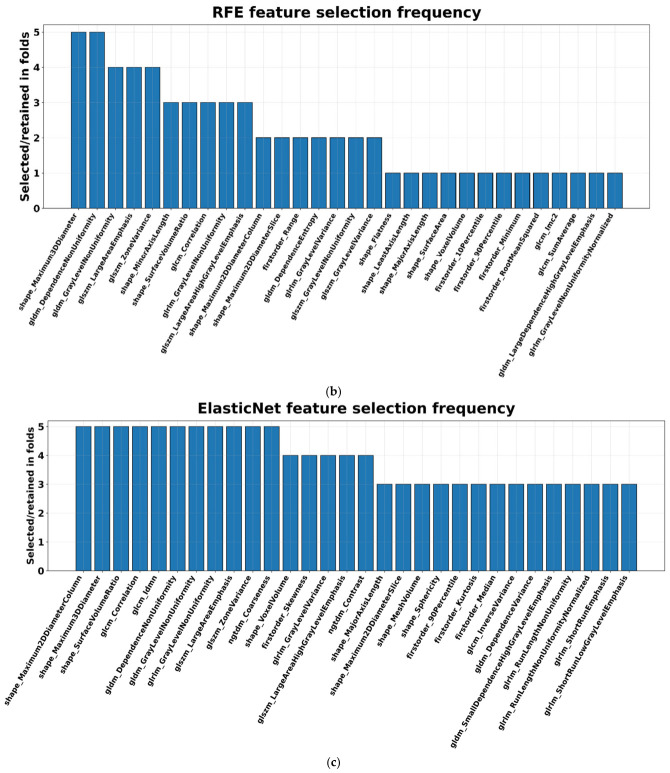

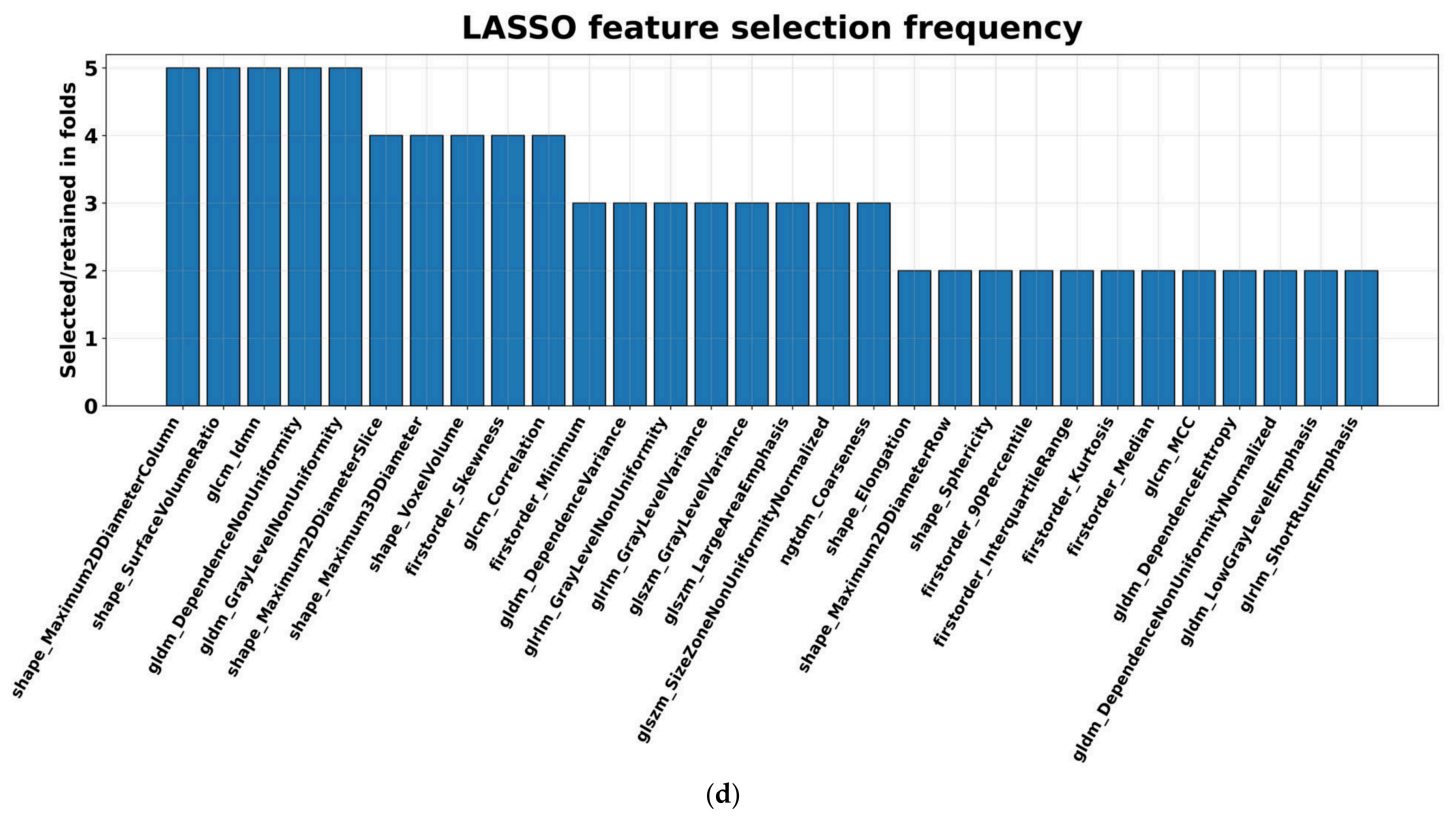
Feature selection and retention frequency analysis under patient-level five-fold cross-validation. The bar plots show the frequency with which radiomic features were retained or selected across the five patient-level cross-validation folds using (**a**) correlation-based filtering at the |ρ| ≥ 0.8 threshold, (**b**) RFE, (**c**) ElasticNet, and (**d**) LASSO. The results indicate that better-performing reduced-feature models retained a combination of shape-based, first-order, and texture-based radiomic descriptors, suggesting that both lymph node morphology and internal gray-level heterogeneity contributed to model discrimination.

Feature selection frequency analysis was performed to examine the stability of retained radiomic features across patient-level five-fold cross-validation for the better-performing feature reduction strategies. The corresponding bar plots are presented in Figure 2, where correlation-based filtering at the |ρ| ≥ 0.8 threshold is shown in Figure 2a, RFE in Figure 2b, ElasticNet in Figure 2c, and LASSO in Figure 2d.

As shown in Figure 2a, correlation-based filtering at the |ρ| ≥ 0.8 threshold demonstrated a highly stable retention pattern across folds. Most retained features appeared in all five folds, indicating that the reduced feature set consistently preserved non-redundant radiomic descriptors. The retained features included shape-based descriptors such as shape_Elongation, shape_Flatness, shape_MajorAxisLength, shape_Sphericity, and shape_SurfaceVolumeRatio, together with first-order and texture features from the GLCM, GLDM, GLSZM, and NGTDM families.

The RFE-based analysis, shown in Figure 2b, produced a more compact and selective feature pattern. The most frequently retained features were shape_Maximum3DDiameter and gldm_DependenceNonUniformity, each selected in all five folds. These were followed by gldm_GrayLevelNonUniformity, glszm_LargeAreaEmphasis, and glszm_ZoneVariance, which were retained in four folds. This pattern suggests that RFE prioritized a limited subset of features representing both lymph node morphology and gray-level texture heterogeneity.

ElasticNet retained a broader and more stable feature set, as shown in Figure 2c. Several features were selected in all five folds, including shape_Maximum2DDiameterColumn, shape_Maximum3DDiameter, shape_SurfaceVolumeRatio, glcm_Correlation, glcm_Idmn, gldm_DependenceNonUniformity, gldm_GrayLevelNonUniformity, glrlm_GrayLevelNonUniformity, glszm_LargeAreaEmphasis, glszm_ZoneVariance, and ngtdm_Coarseness. This indicates that ElasticNet preserved complementary information from multiple radiomic families, including shape, gray-level co-occurrence, gray-level dependence, run-length, size-zone, and neighboring gray-tone difference features.

LASSO also demonstrated a stable but more compact selection profile than ElasticNet, as illustrated in Figure 2d. The features shape_Maximum2DDiameterColumn, shape_SurfaceVolumeRatio, glcm_Idmn, gldm_DependenceNonUniformity, and gldm_GrayLevelNonUniformity were selected in all five folds. Additional shape, first-order, and texture features were retained in three to four folds, suggesting that LASSO identified a compact yet heterogeneous radiomic signature.

Overall, the feature selection frequency analysis indicates that patient-level classification performance was supported by a combination of morphologic and texture-based radiomic information rather than by shape descriptors alone. Shape-related features remained important, particularly diameter-, volume-, and surface-related descriptors; however, texture features reflecting gray-level dependence, non-uniformity, size-zone patterns, and local intensity relationships were also repeatedly selected. These findings suggest that the differentiation of CA-LAP and Sarcoidosis-LAP relies on complementary radiomic information capturing both lymph node morphology and internal gray-level heterogeneity.

A comparative evaluation of the best-performing models obtained using different feature selection and dimensionality reduction strategies is summarized in Table 6. Overall, reduced-feature and component-based approaches generally outperformed the model trained with all 107 radiomic features under patient-level five-fold cross-validation. The highest ROC–AUC was achieved by the LASSO-based model, with an AUC of 0.9108 using 26 selected radiomic features. PCR and RFE also demonstrated strong discriminative performance, both reaching an AUC of 0.8942, while ElasticNet achieved the highest accuracy and F1-score, with values of 81.20% and 81.56%, respectively. ElasticNet, PLS, and RFE showed the highest recall value of 87.00%, indicating improved sensitivity for identifying the positive class. In contrast, the all-feature neural network model achieved a lower AUC of 0.8142, suggesting that the use of the complete high-dimensional feature set did not provide the most favorable performance under patient-level validation. These findings indicate that feature selection and dimensionality reduction improved the balance between model performance, compactness, and interpretability, with LASSO and ElasticNet providing particularly favorable results.

### 3.2. Clinical and Radiomic Feature Integration Analysis

Clinical variables may have an important influence on the differentiation of lung cancer-related lymphadenopathy and sarcoidosis-related lymphadenopathy, particularly because age, sex, and smoking status are clinically relevant factors for these conditions. Therefore, evaluating radiomic models alone may not be sufficient to determine whether radiomic features provide additional or complementary diagnostic information beyond routinely available clinical characteristics. To address this issue, clinical-only and combined clinical–radiomic models were developed using age, sex, and smoking status. All models were evaluated under the same patient-level five-fold cross-validation framework used for the radiomics-only analyses.

The comparative results are presented in Table 7. The clinical-only reference model achieved high discriminative performance, with an accuracy of 94.92% and an ROC–AUC of 0.9733, indicating that the clinical variables carried substantial diagnostic information in this cohort. The best combined clinical–radiomic model achieved an accuracy of 97.78% and an ROC–AUC of 1.0000, corresponding to a modest improvement over the clinical-only reference model. Compared with their radiomics-only counterparts, most combined clinical–radiomic models showed improved performance after clinical variables were incorporated into the candidate feature space. The highest overall performance was obtained by the combined correlation-filtered ensemble model.

Notably, for several feature selection methods, the integration of clinical variables resulted in a more compact selected variable subset while improving classification performance. This pattern was particularly evident for LASSO, ElasticNet, and RFE. For example, the radiomics-only LASSO model selected an average of 25.6 features and achieved an accuracy of 78.98% and an ROC–AUC of 0.9108, whereas the combined LASSO model selected an average of 9.6 variables and improved the accuracy to 91.79% and the ROC–AUC to 0.9667. This suggests that clinical variables reduced redundancy within the radiomic feature space and contributed to a more parsimonious predictive representation.

Overall, these findings indicate that baseline clinical variables carried substantial diagnostic weight in this specific cohort. This highly pronounced performance of the clinical-only model is primarily driven by the distinct demographic cleavage between the two disease groups, where lung cancer patients were significantly older and had a higher prevalence of smoking positivity. In clinical oncology, age and smoking status are already heavily weighted, non-linear risk factors that naturally create a high baseline separation in a non-matched cohort. Consequently, the incremental benefit of adding CT-derived radiomic features appeared statistically modest because the clinical ceiling was already exceptionally high. Rather than diminishing the value of quantitative imaging, these results suggest that radiomics functions as a narrow-spectrum complementary tool rather than a broad-spectrum replacement for clinical history. The fact that combined models still managed to perfect the AUC metric and drastically downsize the required radiomic feature subset (e.g., from 25.6 down to 9.6 features in LASSO) proves that radiomics strips away phenotypic noise and offers a more parsimonious predictive representation when aligned with clinical ground truths.

### 3.3. One-Node-per-Patient Sensitivity Analysis

To further evaluate whether model performance was influenced by patients contributing multiple lymph nodes, a one-node-per-patient sensitivity analysis was performed. Because no predefined clinical priority or anatomical hierarchy was available for selecting a single representative lymph node, one lymph node was randomly selected from each patient, and this procedure was repeated 100 times. In each repetition, radiomics-only, clinical-only, and combined clinical–radiomic models were evaluated using the same patient-level five-fold cross-validation framework.

The results of this sensitivity analysis are summarized in Table 8. The radiomics-only LASSO model showed reduced performance in the one-node-per-patient setting, with an accuracy of 60.83 ± 1.65% and an ROC–AUC of 0.6739 ± 0.0220. In contrast, the clinical-only ensemble model retained high discriminative performance, with an accuracy of 94.84 ± 0.24% and an ROC–AUC of 0.9946 ± 0.0016. The combined clinical–radiomic models also showed high discriminative performance, although they did not exceed the clinical-only reference model in this sensitivity analysis. The combined LASSO model achieved an accuracy of 92.64 ± 0.76% and an ROC–AUC of 0.9726 ± 0.0055, while the combined correlation-filtered ensemble model achieved an accuracy of 91.79 ± 0.82% and an ROC–AUC of 0.9739 ± 0.0055. These findings suggest that the observed performance patterns were not solely attributable to unequal lymph-node contribution across patients. They also indicate that baseline clinical variables retained substantial discriminative information in the one-node-per-patient setting.

### 3.4. Exploratory Interobserver Radiomic Feature Stability Analysis

An exploratory interobserver radiomic feature stability analysis was performed using 30 matched cases independently measured by two radiologists. In this analysis, 107 radiomic features were evaluated using ICC(2,1). The log1p-transformed ICC analysis showed a mean ICC of 0.621 and a median ICC of 0.619. Overall, 77 features showed ICC ≥ 0.50, and 18 features showed ICC ≥ 0.75, suggesting that a subset of radiomic features demonstrated moderate-to-good interobserver stability under the available matched-measurement conditions (Table 9). Because exact nodal-location information was not consistently available in the second measurement set, these findings were interpreted as exploratory rather than definitive node-level reproducibility results.

## 4. Discussion

Mediastinal and hilar lymphadenopathy is a common finding in thoracic imaging and requires comprehensive evaluation in the differential diagnosis of reactive, inflammatory, and malignant causes. Chest radiography and computed tomography (CT) are widely used for lymph node evaluation, with CT providing detailed information on nodal size, shape, margins, density, and enhancement characteristics [22]. However, conventional imaging findings such as nodal size, calcification, or necrosis are often insufficient for reliably differentiating sarcoidosis from malignant lymph node involvement. Similarly, although FDG-PET/CT has high sensitivity in suspected pulmonary sarcoidosis, increased FDG uptake may be observed in both granulomatous and malignant processes, limiting specificity and requiring interpretation together with clinical, laboratory, and histopathological findings [23]. These limitations support the need for quantitative imaging approaches that may complement conventional assessment.

Radiomic approaches have shown value in lung cancer diagnosis, differential diagnosis, histological subtype prediction, and mediastinal lymph node metastasis assessment [24,25,26,27,28,29,30,31]. In granulomatous diseases, PET/CT-based radiomic studies have also suggested potential utility in differentiating tuberculosis, sarcoidosis, and lymphoma-related lymphadenopathy [32,33,34]. However, most previous studies have focused on primary tumors, metastatic lymph node prediction, or comparisons involving lymphoma or tuberculosis. To our knowledge, direct evidence on thoracic CT-derived radiomic features for differentiating mediastinal lymphadenopathy associated with lung cancer from that associated with sarcoidosis remains limited. In this context, the present study provides preliminary patient-level cross-validation evidence that CT-derived radiomic features may have discriminative potential for this clinically challenging differential diagnosis.

In the present cohort, traditional size-based parameters, including both short-axis and long-axis lymph node diameters, did not show statistically significant differences between the sarcoidosis and lung cancer groups. The mean short-axis diameter was 18.55 ± 3.54 mm in the sarcoidosis group and 17.40 ± 3.15 mm in the lung cancer group, while the mean long-axis diameter was 30.95 ± 7.62 mm and 28.07 ± 6.66 mm, respectively. These differences were not statistically significant, with *p*-values of 0.122 and 0.065. Consistently, size-based ROC analysis showed poor discriminative performance, with AUC values of 0.589 and 0.606. These findings support the limitation of conventional size-based assessment in this differential diagnosis. In contrast, radiomic models achieved higher discriminative performance, particularly after feature selection or dimensionality reduction, suggesting that CT-derived radiomic features may capture information beyond simple nodal size.

In the patient-level validation framework, the model trained with all 107 radiomic features did not provide the most favorable performance. The best AUC among all-feature classifiers was obtained by the neural network model, with an ROC–AUC of 0.8142, while logistic regression achieved the highest accuracy of 73.5%. After feature reduction, performance generally improved. Correlation-based filtering at the |ρ| ≥ 0.8 threshold retained an average of 34 features and achieved an accuracy of 75.75% and an ROC–AUC of 0.8500. Embedded and regularized methods provided further improvement. LASSO selected an average of 25.6 radiomic features and achieved the highest radiomics-only ROC–AUC of 0.9108, whereas ElasticNet selected 42 features and achieved the highest radiomics-only accuracy of 81.20%. PCR and RFE also showed strong AUC values of 0.8942. These results indicate that compact or regularized feature representations were more effective than the full high-dimensional feature set, likely because they reduced redundancy and overfitting risk while preserving discriminative information.

The feature selection frequency analysis further indicated that model discrimination was not driven by shape-based descriptors alone. Although morphologic features related to nodal diameter, volume, surface characteristics, and sphericity were repeatedly retained, texture-based descriptors from the GLCM, GLDM, GLRLM, GLSZM, and NGTDM families were also frequently selected. These features reflect gray-level dependence, non-uniformity, size-zone distribution, and local intensity relationships. Therefore, differentiation between CA-LAP and Sarcoidosis-LAP appears to rely on complementary information capturing both lymph node morphology and internal gray-level heterogeneity. This finding is important because the conventional size measurements were not significantly different between groups, whereas radiomic models using both shape and texture information showed substantially higher AUC values. Thus, the results support a multi-family radiomic signature rather than a purely shape-dominant feature pattern.

This interpretation is consistent with thoracic radiology practice, in which conventional CT findings such as nodal size, contour, internal density, necrosis, calcification, and enhancement may overlap between granulomatous and malignant lymphadenopathy, limiting the reliability of visual assessment alone. From a pathological perspective, this pattern may reflect different mechanisms of lymph node involvement. Sarcoidosis-related granulomatous inflammation may be associated with relatively homogeneous inflammatory enlargement and better-preserved nodal contours, whereas metastatic infiltration may produce asymmetric growth, capsular distortion, necrotic change, and heterogeneous nodal expansion. These processes may influence shape-related descriptors such as sphericity, volume, surface characteristics, elongation, and flatness, as well as texture features reflecting internal gray-level heterogeneity. However, these interpretations should be considered hypothesis-generating because radiomic features are indirect quantitative descriptors rather than direct histopathological measurements.

The clinical–radiomic integration analysis provided an additional perspective on the diagnostic information contained in clinical and imaging-derived variables. The clinical-only ensemble model, using only age, sex, and smoking status, achieved high performance, with an accuracy of 94.92% and an ROC–AUC of 0.9733. This result indicates that baseline clinical differences between the two groups carried substantial discriminative information. This is consistent with the demographic profile of the cohort, in which the lung cancer group had a significantly higher mean age and a higher frequency of smoking positivity. When clinical variables were incorporated into the candidate feature space together with radiomic features, selected combined models achieved the highest overall performance. The combined correlation-filtered ensemble model reached an accuracy of 97.78% and an ROC–AUC of 1.00, while combined ElasticNet and Boruta also achieved AUC values of 1.0000. These findings suggest that radiomic features may provide complementary information when integrated with clinical variables.

However, the magnitude of improvement over the clinical-only model should be interpreted carefully. The best combined model improved accuracy from 94.92% to 97.78% and ROC–AUC from 0.9733 to 1.00 compared with the clinical-only reference model. Although this represents the highest overall performance, the absolute improvement was modest because the clinical-only model was already highly discriminative. Therefore, the results should not be interpreted as evidence that radiomics substantially outperformed clinical variables. Rather, they suggest that radiomic features may add complementary information in selected model configurations. This interpretation is further supported by the behavior of feature selection models. For example, radiomics-only LASSO selected an average of 25.6 features and achieved an ROC–AUC of 0.9108, whereas combined LASSO selected a smaller subset of 9.6 variables and improved the ROC–AUC to 0.9667. Similarly, RFE decreased from 14 selected radiomic features to 3 variables after clinical integration, while maintaining a high ROC–AUC of 0.9333. This pattern suggests that clinical variables may reduce redundancy within the radiomic feature space and allow more parsimonious predictive representations.

The one-node-per-patient sensitivity analysis further addressed the potential influence of unequal lymph-node contribution across patients. This analysis was important because the full dataset included 204 lymph nodes, corresponding to an average of approximately 5.5 lymph nodes per patient. Although all lymph nodes from the same patient were kept within the same cross-validation fold, patients contributing more lymph nodes could still have had a greater influence on model evaluation. When one lymph node was randomly selected per patient over 100 repetitions, the radiomics-only LASSO model showed a marked reduction in performance, with an accuracy of 60.83 ± 1.65% and an ROC–AUC of 0.6739 ± 0.0220. In contrast, the clinical-only ensemble model retained high performance, with an accuracy of 94.84 ± 0.24% and an ROC–AUC of 0.9946 ± 0.0016. Combined clinical–radiomic models also remained strong, with ROC–AUC values of 0.9726 ± 0.0055 for combined LASSO and 0.9739 ± 0.0055 for the combined correlation-filtered ensemble model. These findings suggest that the main performance trends were unlikely to be explained solely by unequal lymph-node contribution across patients; however, the reduced performance of radiomics-only models in the one-node-per-patient sensitivity analysis indicates that radiomic signatures may be sensitive to representative node selection, particularly when only a single lymph node is available per patient.

The exploratory interobserver stability analysis also supports a cautious interpretation of feature-level robustness. Using 30 matched cases, the log1p-transformed ICC analysis showed a mean ICC of 0.621 and a median ICC of 0.619. Overall, 77 of 107 features showed ICC ≥ 0.50, while 18 features showed ICC ≥ 0.75. These results indicate that a subset of radiomic features demonstrated moderate-to-good interobserver stability, but not all features were robust to measurement variability. Moreover, exact node-level correspondence could not be fully confirmed because nodal-location information was not consistently available in the second measurement set. Therefore, the ICC findings should be interpreted as supportive and exploratory rather than definitive node-level reproducibility evidence. This issue is particularly relevant for radiomic studies based on manual segmentation, where differences in ROI delineation may influence both morphologic and texture-based feature values.

In summary, CT-derived radiomic analysis may provide complementary diagnostic information when conventional imaging findings are insufficient. The lack of significant differences in short- and long-axis diameters between groups suggests that traditional measurements alone may be inadequate for reliable differentiation. Radiomics-only models, particularly LASSO and ElasticNet, showed stronger discriminative performance than conventional size parameters and the full unreduced feature set. In addition, combined clinical–radiomic models achieved the highest overall performance, suggesting potential complementarity between clinical and imaging-derived quantitative information. Nevertheless, clinical variables were highly informative, the improvement over clinical-only models was modest, and radiomics-only performance decreased in the one-node-per-patient sensitivity analysis. Therefore, these findings should be interpreted as preliminary evidence supporting the potential role of radiomics as a non-invasive decision-support tool rather than as a replacement for histopathological confirmation. Larger multicenter studies with independent external validation, standardized segmentation protocols, representative node selection strategies, and clinically balanced cohorts are required before routine clinical implementation can be recommended.

### Limitations

The main limitations of this study include its retrospective, single-center design, the relatively limited number of patients, and the absence of an external validation cohort. Although patient-level five-fold cross-validation was used for internal validation, this approach cannot fully replace validation on an independent dataset acquired from different scanners, institutions, or patient populations. Therefore, the reported performance metrics should be considered preliminary and hypothesis-generating, particularly given the high-dimensional nature of radiomic features despite the use of feature selection and dimensionality reduction strategies.

Another critical limitation is the absence of a propensity score-matched clinical cohort. Because this was a retrospective study reflecting real-world institutional data, the baseline clinical differences (age and smoking behavior) between the sarcoidosis and lung cancer cohorts were structurally skewed. This clinical imbalance created an artificially high performance ceiling for the clinical-only model, masking the potential independent diagnostic power of the radiomic signatures. To definitively isolate the mathematical value of CT radiomics in differentiating these two entities, future prospective or multi-center studies must utilize strict demographic matching protocols—such as age- and smoking-status matching—to eliminate clinical confounding factors. Validating these models on a clinically balanced cohort where demographics overlap entirely will be the next crucial step before any routine clinical implementation can be recommended.

Although patient-level cross-validation ensured that lymph nodes from the same patient were not shared between training and validation folds, the dataset still included multiple lymph nodes from some patients. In total, 204 lymph nodes were obtained, corresponding to an average of approximately 5.5 lymph nodes per patient. Therefore, unequal lymph-node contribution and residual within-patient correlation may still influence performance estimates. To further address this issue, a one-node-per-patient sensitivity analysis was performed by randomly selecting one lymph node from each patient across 100 repetitions. Although this analysis reduced the potential influence of unequal lymph-node contribution, the reduced performance of the radiomics-only LASSO model in this setting indicates that radiomics-only findings may be sensitive to representative node selection. Future studies should validate these findings using larger datasets with standardized representative-node selection or robust patient-level aggregation strategies.

Another limitation is related to segmentation reproducibility. Although an exploratory interobserver radiomic feature stability analysis was performed using 30 matched cases independently measured by two radiologists, this analysis was based on a limited subset of cases. In addition, exact node-level correspondence could not be fully confirmed because nodal-location information was not consistently available in the second measurement set. Therefore, the ICC findings should be interpreted as supportive but exploratory evidence rather than definitive node-level interobserver validation. Future studies should include standardized node-level segmentation protocols, confirmed nodal correspondence, and larger interobserver reproducibility analyses.

Finally, the exclusion of non-diagnostic cases may have introduced selection bias. However, this approach was necessary to ensure reliable radiomic feature extraction and to prevent the inclusion of noisy or incomplete feature vectors. Future prospective multicenter studies with independent external validation, balanced clinical characteristics, patient-level outcome assessment, and reproducibility analyses are warranted to confirm the robustness and clinical applicability of these findings.

## 5. Conclusions

In conclusion, this study suggests that CT-derived radiomic features may provide complementary diagnostic information for the non-invasive differentiation of mediastinal lymphadenopathy associated with lung cancer and sarcoidosis. Under patient-level five-fold cross-validation, reduced-feature and regularized radiomic models showed more favorable performance than the full high-dimensional radiomic feature set. In particular, the LASSO-based model achieved the highest ROC–AUC among radiomics-only models, whereas ElasticNet achieved the highest accuracy, recall, and F1-score. Feature selection frequency analysis indicated that model discrimination was supported by a combination of shape-based, first-order, and texture-based radiomic descriptors rather than by shape-based features alone.

Clinical–radiomic integration analysis showed that age, sex, and smoking status were highly informative in this cohort, while selected combined clinical–radiomic models showed numerically higher performance. However, the strong performance of the clinical-only model and the reduced radiomics-only performance in the one-node-per-patient sensitivity analysis indicate that the independent incremental value of radiomic features should be interpreted cautiously. Therefore, CT-derived radiomics should be considered a potential complementary decision-support approach rather than a replacement for clinical assessment or histopathological confirmation.

The exploratory interobserver analysis also indicated that radiomic feature values may be influenced by segmentation and measurement variability; therefore, reproducibility should be confirmed using standardized node-level segmentation protocols in future studies. Given the retrospective single-center design, the relatively limited number of patients, and the absence of independent external validation, these findings should be considered preliminary. Future multicenter studies with independent validation cohorts, clinically balanced populations, standardized representative-node selection strategies, and reproducibility analyses are required before routine clinical implementation can be recommended.

## Figures and Tables

**Table 1:** Baseline demographic and clinical characteristics of the study population. The lung cancer group had a significantly higher mean age than the sarcoidosis group (*p* < 0.001). Smoking positivity was more frequent in the lung cancer group, although this finding was at the threshold of statistical significance (*p* = 0.050), whereas the sex distribution did not differ significantly between groups (*p* = 0.170). No statistically significant differences were observed in mean lymph node short-axis (*p* = 0.122) or long-axis diameters (*p* = 0.065) between the two groups.

Characteristic	Sarcoidosis	Lung Cancer	*p*-Value
**Patients, *n* (%)**	18 (48.6)	19 (51.4)	—
**Lymph nodes, *n* (%)**	102 (50.0)	102 (50.0)	—
**Lymph nodes per patient, mean ± SD**	5.67 ± 2.43	5.37 ± 2.54	0.717
**Lymph nodes per patient, median**	6.0	5.0	—
**Sex, *n* (%)**			0.170
Male	10 (55.6)	15 (78.9)	
Female	8 (44.4)	4 (21.1)	
**Age, years, mean ± SD**	39.89 ± 6.30	64.05 ± 8.12	<0.001
**Smoking status, *n* (%)**			0.050
Positive	6 (33.3)	13 (68.4)	
Negative	12 (66.7)	6 (31.6)	
**Short-axis diameter, mm, mean ± SD**	18.55 ± 3.54	17.40 ± 3.15	0.122
**Long-axis diameter, mm, mean ± SD**	30.95 ± 7.62	28.07 ± 6.66	0.065

**Table 2:** Classification performance of machine learning models using all 107 radiomic features without dimensionality reduction.

Model	Accuracy (%)	Recall (%)	Specificity (%)	F1-Score (%)	Precision (%)	ROC-AUC
**Discriminant**	55.1	73.0	38.3	61.3	54.0	0.5475
**Tree**	59.9	63.0	61.7	60.1	66.7	0.6533
**SVM**	65.5	68.0	61.7	65.0	63.0	0.6775
**kNN**	52.6	55.0	50.0	48.7	44.0	0.7042
**Ensemble**	71.7	74.0	71.7	70.0	70.3	0.7667
***LR***	73.5	74.0	75.0	72.2	75.3	0.8042
**Neural Network**	62.0	67.0	53.3	63.0	61.8	0.8142

**Table 3:** Classification performance of the best-performing machine learning model selected for each correlation-based feature filtering configuration.

Feature Set (n)	Correlation Threshold	Best Model	Number of Features	Accuracy	Recall	Specificity	Precision	F1-Score	ROC-AUC
**17**	|ρ| ≥ 0.6	Ensemble	17.0	68.9	74.0	66.7	73.7	67.0	0.8433
**22**	|ρ| ≥ 0.7	LR	21.8	72.8	75.0	70.0	62.4	67.3	0.8175
**52**	|ρ| ≥ 0.9	Neural Network	52.4	72.9	74.0	71.7	80.3	71.0	0.8425
**34**	|ρ| ≥ 0.8	Ensemble	34.0	75.8	79.0	73.3	81.3	73.8	0.8500

**Table 4:** Effect of Selected Radiomic Features on Classification Performance Using LASSO, ElasticNet, and Boruta Feature Selection Methods.

Method	Number of Radiomic Features	Accuracy	Recall	Specificity	Precision	F1-Score	ROC-AUC
**Boruta**	8	61.3	63.0	60.0	61.0	60.8	0.7250
**ElasticNet**	42	81.2	87.0	76.7	80.3	81.6	0.8858
**LASSO**	26	79.0	83.0	76.7	80.3	78.0	0.9108

**Table 5:** Classification performance of dimensionality reduction methods under patient-level five-fold cross-validation.

Method	Number of Components	Accuracy (%)	Recall (%)	Specificity (%)	Precision (%)	F1-Score (%)	ROC-AUC
**PLS**	5 components	75.48	87.00	63.33	75.76	78.32	0.8192
**PCR**	19 components	79.70	77.00	81.67	81.00	77.78	0.8942

**Table 6:** Comparison of the best classification performances obtained using different feature selection and dimensionality reduction strategies.

Method	Number of Features/Components	Accuracy (%)	Recall (%)	Specificity (%)	Precision (%)	F1-Score (%)	AUC
**Boruta**	8	61.25 (54.56–67.94)	63.00	60.00	61.00	60.78	0.7250 (0.6557–0.7943)
**All features + Neural Network**	107	62.04 (55.37–68.71)	67.00	53.33	61.76	62.97	0.8142 (0.7553–0.8731)
**PLS**	5 components	75.48 (69.58–81.38)	87.00	63.33	75.76	78.32	0.8192 (0.7610–0.8774)
**Correlation-based filtering |ρ| ≥ 0.8 + Ensemble**	34	75.75 (69.86–81.64)	79.00	73.33	81.33	73.78	0.8500 (0.7966–0.9034)
**ElasticNet**	42	81.20 (75.82–86.58)	87.00	76.67	80.33	81.56	0.8858 (0.8389–0.9327)
**PCR**	19 components	79.70 (74.19–85.21)	77.00	81.67	81.00	77.78	0.8942 (0.8491–0.9393)
**RFE**	14	77.62 (71.90–83.34)	87.00	66.67	76.67	80.33	0.8942 (0.8491–0.9393)
**LASSO**	26	78.98 (73.39–84.57)	83.00	76.67	80.33	77.98	0.9108 (0.8693–0.9523)

**Table 7:** Comparative performance of clinical-only, radiomics-only, and combined clinical–radiomic models under patient-level five-fold cross-validation.

Approach/Method	Radiomics-Only				Combined			
	Model	Number of Features/Components	Acc (%)	AUC	Model	Number of Features/Components	Acc (%)	AUC
**Clinical-only reference**	—	—	—	—	Ensemble	3	94.92 ± 8.75	0.9733 ± 0.0476
**All features**	Neural Network	107	62.04 ± 9.59	0.814 ± 0.201	Ensemble	110	97.78 ± 6.17	1.00 ± 0.00
**Correlation-based filtering**	Correlation |ρ| ≥ 0.8 + Ensemble	34	75.75 ± 22.14	0.850 ± 0.277	Correlation 0.7 + Ensemble	25	97.78 ± 6.17	1.00 ± 0.00
**LASSO**	LASSO	26	78.98 ± 10.51	0.910 ± 0.153	LASSO	10	91.79 ± 15.65	0.966 ± 0.092
**ElasticNet**	ElasticNet	42	81.20 ± 6.56	0.885 ± 0.160	ElasticNet	1	92.14 ± 14.16	1.00 ± 0.00
**Boruta**	Boruta	8	61.25 ± 23.21	0.725 ± 0.208	Boruta	7	94.92 ± 8.75	1.00 ± 0.00
**PCR**	PCR	19	79.70 ± 18.23	0.894 ± 0.159	PCR	20	90.28 ± 20.19	0.962 ± 0.104
**PLS**	PLS	5.2	75.48 ± 13.97	0.819 ± 0.118	PLS	12	88.93 ± 23.06	0.954 ± 0.090
**RFE**	RFE	14	77.62 ± 16.10	0.894 ± 0.159	RFE	3	86.71 ± 21.80	0.933 ± 0.119

Note: Clinical-only models used age, sex, and smoking status. Combined clinical–radiomic models incorporated these clinical variables into the candidate feature space together with radiomic features. For correlation-based filtering, only the best-performing configuration is shown for each feature set type. The number of variables/components represents the mean value across five patient-level cross-validation folds.

**Table 8:** One-node-per-patient sensitivity analysis across 100 random repetitions.

Approach	Method	Repetitions	Number of Variables	Accuracy (%)	ROC–AUC
Radiomics-only	LASSO	100	9.10 ± 0.61	60.83 ± 1.65	0.6739 ± 0.0220
Clinical-only	Ensemble	100	3.00 ± 0.00	94.84 ± 0.24	0.9946 ± 0.0016
Combined clinical–radiomic	LASSO	100	8.67 ± 0.55	92.64 ± 0.76	0.9726 ± 0.0055
Combined clinical–radiomic	Correlation 0.7 + Ensemble	100	22.34 ± 0.30	91.79 ± 0.82	0.9739 ± 0.0055

Note: One lymph node was randomly selected from each patient, and the procedure was repeated 100 times. Values are reported as mean ± 95% confidence interval across repetitions. Variables/components represent the mean number selected or retained across repetitions.

**Table 9:** Exploratory interobserver radiomic feature stability analysis using ICC(2,1). Note: ICC, intraclass correlation coefficient. The analysis was performed on matched cases with available second-radiologist measurements. Because nodal-location information was not consistently available in the second measurement set, this analysis was interpreted as exploratory.

Analysis	Matched Cases	Features Evaluated	Mean ICC	Median ICC	Features with ICC ≥ 0.50	Features with ICC ≥ 0.75
**Log1p-transformed ICC analysis**	30	107	0.621	0.619	77	18

## Data Availability

The data presented in this study are available on reasonable request from the corresponding author. The data are not publicly available due to patient privacy and institutional ethical restrictions.

## Abbreviations

The following abbreviations are used in this manuscript:

AUC	Area Under the Curve
CA-LAP	Cancer-Associated Lymphadenopathy
CV	Cross-Validation
CT	Computed Tomography
EBUS	Endobronchial Ultrasound
GLCM	Gray-Level Co-occurrence Matrix
GLRLM	Gray-Level Run Length Matrix
GLSZM	Gray-Level Size Zone Matrix
kNN	k-Nearest Neighbor
LASSO	Least Absolute Shrinkage and Selection Operator
Boruta	Boruta Feature Selection Algorithm
ElasticNet	Elastic Net Regularization
NN	Neural Network
PCR	Principal Component Regression
PLS	Partial Least Squares
RFE	Recursive Feature Elimination
ROC	Receiver Operating Characteristic
ROI	Region of Interest
SVM	Support Vector Machine
LR	Logistic Regression
